# Supplementary material for: A whole-animal phenotypic drug screen identifies suppressors of atherogenic lipoproteins
Source: eLife. 2026 Aug 27;14:RP105314. doi: 10.7554/eLife.105314 (PMC13521566; doi:10.7554/eLife.105314)
Supplement: Supplementary file 2 [file elife-105314-code1.zip › JHDL_primary_result/MEDROXYPROGESTERONE ACETATE/Medroxyprogesterone acetate_JHDL_14_027_002_2019_08_30_10_37.pdf]

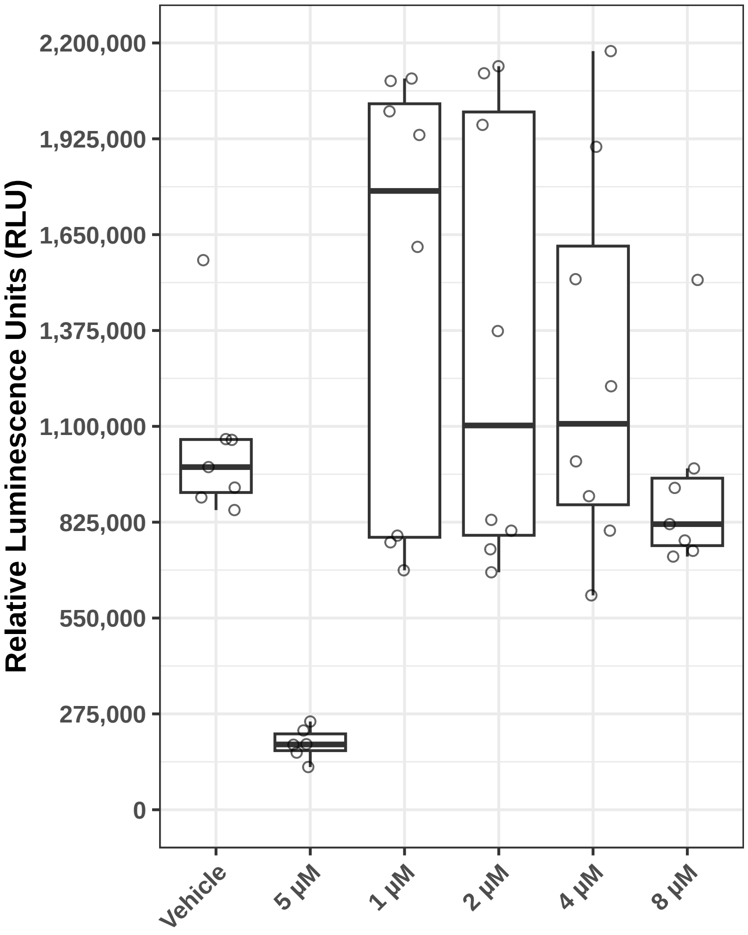
Relative Luminescence Units (RLU)
2,200,000
1,925,000
1,650,000
1,375,000
1,100,000
825,000
550,000
275,000
0
Vehicle
5 µM
1 µM
2 µM
4 µM
8 µM
